# Immunogenicity of an adalimumab biosimilar, FKB327, and its reference product in patients with rheumatoid arthritis

**DOI:** 10.1111/1756-185X.13951

**Published:** 2020-08-27

**Authors:** Rieke Alten, Colin Markland, Malcolm Boyce, Kazuki Kawakami, Rafael Muniz, Mark C. Genovese

**Affiliations:** ^1^ University Medicine Berlin Berlin Germany; ^2^ NDA Group Leatherhead UK; ^3^ Hammersmith Medicines Research London UK; ^4^ Fujifilm Kyowa Kirin Biologics Tokyo Japan; ^5^ Mylan Inc Canonsburg PA USA; ^6^ Stanford University Palo Alto CA USA

**Keywords:** adalimumab, biosimilars, immunogenicity, rheumatoid arthritis, switching, TNF inhibitors

## Abstract

**Aim:**

This study, FKB327‐003, is a phase 3, open‐label extension (OLE) study comparing the long‐term immunogenicity of an adalimumab biosimilar, FKB327 (F), with the reference product (RP).

**Methods:**

In the OLE, patients completing 24 weeks of an initial randomized, double‐blind (DB) study (Period 1) with clinical response and no safety concerns were rerandomized to F or RP, so that two‐thirds of patients remained on the same treatment and one‐third switched to the alternate treatment for weeks 24 through 54 (OLE weeks 0‐30; Period 2), then all received F through week 100 (OLE week 76; Period 3). Treatment sequences were F‐F‐F (no switch), RP‐F‐F and RP‐RP‐F (single switch), and F‐RP‐F (double switch). Patients who entered the OLE study were evaluated for immunogenicity across switching sequences.

**Results:**

The proportion of patients with positive antidrug antibody (ADA) status at the end of Period 1 was 61.7% and 60.0% for F and RP, respectively. The proportion of patients with positive ADA status did not increase throughout Period 1, and was similar for F and RP at all time points. At the end of Period 3, the proportion of patients with positive ADA status was lower in all treatment sequences, at 51.1%, 54.4%, 48.1%, and 42.5% for F‐F‐F, F‐RP‐F, RP‐F‐F, and RP‐RP‐F, respectively.

**Conclusion:**

The RP and F showed comparable immunogenicity characteristics after long‐term administration. Development of ADAs with the RP and F was similar, and was not impacted by switching and double switching between F and RP treatment.

## 1 INTRODUCTION

Tumor necrosis factor (TNF)‐alpha inhibitors are the most common type of biologics used for treating patients with rheumatoid arthritis (RA) who do not respond to methotrexate (MTX) or other disease‐modifying antirheumatic drugs (DMARDs). TNF‐alpha inhibitors include infliximab, certolizumab pegol, etanercept, golimumab, and adalimumab.^1^ Adalimumab is approved globally for treating RA, juvenile idiopathic arthritis, psoriatic arthritis, ankylosing spondylitis, Crohn's disease, ulcerative colitis, plaque psoriasis, hidradenitis suppurativa, and uveitis.^2^

Patent protection of adalimumab is approaching its expiration date, which will allow biosimilars to be marketed and made available to patients. Biosimilars are defined as biological products that are highly similar to, and have no clinically meaningful differences from, the existing US Food and Drug Administration‐ and European Medicines Agency‐approved reference products (RPs).^3^

FKB327 (adalimumab) was developed as a biosimilar product of adalimumab. In a randomized, double‐blind (DB) study conducted to compare the pharmacokinetics (PK), safety, tolerability, and immunogenicity of FKB327 with both European Union‐approved and US‐licensed RP in healthy adult subjects, FKB327 demonstrated similar PK, safety, tolerability, and immunogenicity to EU‐ and US‐RP, following a single subcutaneous dose of FKB327 or the RP.^4^

Biologic therapies have unique structures that can induce immune responses, which may lead to the development of therapy‐limiting adverse events (AEs).^5^ Antidrug antibodies (ADAs) have been implicated as contributors to increased risk for AEs and treatment failure in patients who are treated with biologics.^6^ Data suggest that most patients treated with natalizumab and adalimumab develop ADAs, with the majority developing within the first 6 months of treatment.^7^ Notably, the presence of ADAs alone does not guarantee an impaired clinical response. Therapeutic levels of active drug may still be present as long as the ADA levels are not sufficient to bind all therapeutic antibodies, as has been demonstrated in patients treated with natalizumab who exhibit an ADA response.^8^ The long‐term safety and immunogenicity of FKB327 following repeated dosing in patients with moderate‐to‐severe RA are currently unknown.

To begin answering these questions, a DB phase 3 study and a long‐term open‐label extension (OLE) study were designed to compare the safety, efficacy, and immunogenicity of FKB327 with the US‐approved RP in patients with RA. Preliminary results through 54 weeks of treatment have been previously published.^9^ Therefore, the aim of the current study was to compare the long‐term immunogenicity and safety of FKB327 with the RP in patients with RA out to 104 weeks, including patients who single‐ and double‐switched treatments between FKB327 and the RP. The immunogenicity of the RP and FKB327 was examined across the DB and long‐term OLE studies. Additional objectives included the following: (a) comparing the proportion of patients developing ADAs with long‐term treatment; (b) comparing the PK of long‐term treatment; and (c) evaluating changes in PK and immunogenicity in patients who were single‐ or double‐switched between the RP and FKB327.

## 2 METHODS

### 2.1 Study design

The initial part of the study design of the DB and the OLE has been previously described.^9^ The FKB327‐002 DB study (National Institutes of Health [NIH] US National Library of Medicine, NCT02260791/EudraCT Number: 2014‐000109‐11, https://clinicaltrials.gov/ct2/show/NCT02260791) was a multicenter, phase 3 study in which patients were randomized 1:1, based on prior biological treatment and screening disease activity, to receive FKB327 or the RP (each dosed at 40 mg subcutaneously) every other week for 22 weeks (Period 1); all patients received MTX 10‐25 mg per week for at least 8 weeks prior to screening.

**Figure 1:**
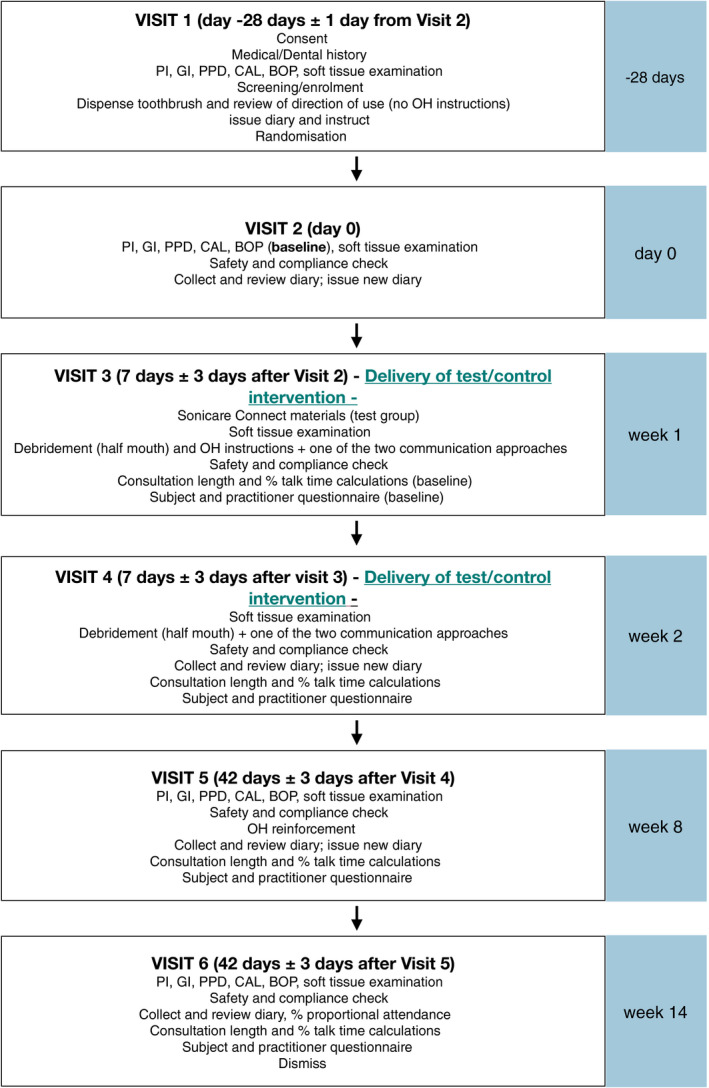
Study design. DB, double‐blind study; EOW, every other week; F, FKB327; OLE, open‐label extension study; R, randomized; RA, rheumatoid arthritis; RP, reference product (US‐approved); SC, subcutaneous; w, week. 730 patients were initially enrolled in the study, but 2 patients dropped out prior to randomization and receipt of study drug

At week 24, patients were eligible to enter the OLE study (FKB327‐003; NIH US National Library of Medicine, NCT02405780/EudraCT Number: 2014‐000110‐61, https://clinicaltrials.gov/ct2/show/NCT02405780) and were rerandomized to receive FKB327 or the RP from week 0 to 30 (Period 2; week 24 to 54 from start of the DB study), with two‐thirds of patients remaining on the same treatment as in the DB study and one‐third of patients switching to the alternate treatment (Figure 1). In Period 3, all patients received FKB327 from week 30 to 76 (week 54‐100 from start of the DB study), followed by a 4‐week follow‐up period (week 80; week 104 from start of the DB study). From the start of the DB study, patients received FKB327 and/or the RP for 100 weeks.

Clinic visits occurred every 4‐12 weeks during Period 2 and every 12 weeks during Period 3. FKB327 (40 mg/0.8 mL adalimumab) was administered via a prefilled plastic syringe with a safety device for single use. US‐licensed RP (40 mg/0.8 mL adalimumab) was administered via a prefilled Type 1 glass syringe during Period 2. In Period 3, during the FKB327 single‐arm treatment phase, the FKB327 autoinjector (AI; 40 mg/0.8 mL adalimumab) was introduced, except to US patients, because of regulatory considerations. FKB327 was manufactured by Kyowa Hakko Kirin Co, Ltd and the RP was US‐licensed Humira, supplied by the sponsor.

Patient groups can be described using the following sequences:

1. F‐F‐F: randomized to FKB327 in Period 1; rerandomized to the same treatment in Period 2; received FKB327 in Period 3 (no switching)
2. RP‐RP‐F: randomized to the RP in Period 1; rerandomized to the same treatment in Period 2; received FKB327 in Period 3 (1 switch)
3. F‐RP‐F: randomized to FKB327 in Period 1; rerandomized to the RP in Period 2; received FKB327 in Period 3 (2 switches)
4. RP‐F‐F: randomized to the RP in Period 1; rerandomized to FKB327 in Period 2; received FKB327 in Period 3 (1 switch).

### 2.2 Patients

Inclusion and exclusion criteria have been previously described.^9^ Briefly, patients were aged ≥18 years with moderate‐to‐severe, inactive, inadequately controlled RA despite MTX management for ≥3 months. At baseline screening for Period 1, patients had ≥6 tender joint count and ≥6 swollen joint count at screening and baseline, and C‐reactive protein ≥10 mg/L. Patients received MTX (10‐25 mg/wk) throughout the study period. Patients had to complete all 24 weeks of procedures in Period 1, with a minimum of 9 study drug doses received and a clinical response to treatment as determined by investigator opinion.

Patients who had evidence of an ongoing severe AE from Period 1, noncompliance with study procedures, or acute infection requiring antibiotic treatment within 2 weeks of week 0 dosing were not eligible for study inclusion in the OLE Period 2 or 3.

All procedures performed in studies involving human participants were in accordance with the ethical standards of the institutional and/or national research committee, the 1964 Helsinki Declaration and its later amendments or comparable ethical standards, and International Conference on Harmonisation Guidelines for Good Clinical Practice. An independent ethics committee or institutional review board for each study center reviewed and approved all study protocols. Written informed consent was obtained from all individual participants included in the study prior to entry.

### 2.3 Immunogenicity assays

Blood samples were collected prior to dosing and at prespecified time points (weeks 12, 24, 30, 54, 76, and 80) to assess adalimumab serum concentration and ADA activity. Serum concentration and ADA testing were conducted at Syneos Health, Princeton, NJ, USA. Immunogenicity was assessed by ADA evaluation (proportion of patients ADA‐positive, ADA titer) using a validated, high‐sensitivity, drug‐tolerant electrochemiluminescence assay with an acid dissociation step (used to improve drug tolerance in serum for multiple‐dosing treatment), using Meso Scale Discovery (MSD) high‐bind plates to detect ADAs against adalimumab as FKB327 or the RP. The confirmation of specificity of these assays used a floating cutoff inhibition point of 28.5%‐35.7%. Titers were classified as negative; below the lower limit of quantification; 1, 4, 16, 64, 256, 1024, 4096, or 16 384; or above the upper limit of quantification. Mean serum trough concentrations of adalimumab were determined via a similar validated, high‐sensitivity electrochemiluminescence method using high‐bind plates coated with TNF‐alpha. For reliable quantification, the lower limit was 100 ng/mL.

ADA neutralizing capability was assessed using MSD plates on an electrochemiluminescence platform, which determined the ability of serum samples to block the binding of ruthenylated adalimumab to immobilized TNF‐alpha on the assay plates. Neutralizing ADA results were reported, noting that circulating drug concentrations exceeded assay drug tolerance limits (>500 ng/mL) when applicable. Samples analyzed in the presence of drug concentrations above assay tolerance limits were deemed inconclusive. If samples tested negative for ADAs in the electrochemiluminescence immunoassay, they were not tested for neutralizing ADAs.

### 2.4 Safety evaluations

Safety was assessed via documentation of AEs, which were evaluated for severity and relationship to study drug. Treatment‐emergent AEs (TEAEs) were summarized using the *Medical Dictionary for Regulatory Activities*. The number of patients experiencing TEAEs by each period and the exposure‐adjusted number of events (number of events divided by patient‐year) was determined. Latent tuberculosis was monitored with QuantiFERON Gold blood tests at weeks 24 and 76. Any patients testing positive for tuberculosis were excluded from the study.

### 2.5 Statistical analyses

Summary statistics, including the number of patients, mean, standard deviation, median, minimum and maximum, were presented for all continuous variables. For categorical variables, per category, the absolute counts (n) and percentages of patients with data, and if appropriate, the number of patients with missing data, were presented. In general, missing data were not imputed.

The primary PK analysis was comprised of 2 mixed models for repeated measures fitted to the log‐transformed serum concentrations at weeks 12, 24, and 30 (during the randomized treatment period), with patients included as a random effect and the following as fixed effects: (a) week, treatment group, and week × treatment group; and (b) week, treatment sequence, and week × treatment sequence.

Due to the potential formation of ADAs, the primary analysis was repeated for the overall treatment period with ADA titer results at the last sampling time point included as an additional covariate, along with an ADA titer × treatment sequence interaction term to test for differences in the effect of ADA activity on the PK data.

All analysis datasets and output were produced by the Biostatistics Department of Quanticate UK Limited, using the SAS^®^ system Version 9.3 (Unicode Support).

## 3 RESULTS

### 3.1 Baseline demographics

In the DB study (Period 1), 730 patients were initially enrolled; however, 2 patients dropped out of the study prior to randomization and receipt of study drug. Therefore, 366 patients were randomized to FKB327 and 362 patients were randomized to the RP (Figure 1). Of these, 333 patients (90.7%) in the FKB327 group and 328 patients (90.4%) in the RP group completed the study. A total of 16 patients (FKB327, 9; RP, 7) did not proceed to the OLE study due to patient preference or investigator opinion. Therefore, 645 patients from 92 sites in 11 countries were enrolled in the OLE, including 242 patients from Europe (Czech Republic, Germany, Poland, Romania, and Spain; 37.5%), 76 from North America (US and Canada; 11.8%), and 327 from the rest of the world (Chile, Peru, Russia, and Ukraine; 50.7%). Of the 324 patients who received FKB327 in Period 1, 216 were randomized to FKB327 (F‐F‐F) and 108 to the RP (F‐RP‐F) in Period 2. Of the 321 patients who received the RP in Period 1, 108 were randomized to FKB327 (RP‐F‐F) and 213 to RP (RP‐RP‐F) in Period 2. Among the patients who completed Period 3, 174 patients did not switch treatments (F‐F‐F), 253 patients were single switched (RP‐RP‐F and RP‐F‐F), and 88 patients were double‐switched (F‐RP‐F). Treatment sequences were well matched based on demographic and disease characteristics (Table 1). The mean age was 52.9 years, with the majority being female (77.7%) and White (85.6%). Overall, 64.8% of patients had received at least 1 DMARD previously, with MTX being most common (43.9%). In all, 18.0% of patients received a prior biologic for RA, and 6.4% of patients received prior anti‐TNF therapy.

**Table 1:** Baseline patient demographics

	F‐F‐F n = 216	F‐RP‐F n = 108	RP‐F‐F n = 108	RP‐RP‐F n = 213	Total N = 645
Age, y					
Mean (SD)	52.7 (12.4)	52.1 (11.4)	52.3 (11.9)	54.0 (12.6)	52.9 (12.2)
Range	18‐85	24‐77	23‐82	21‐93	18‐93
Age range, n (%)					
<65	183 (84.7)	92 (85.2)	96 (88.9)	169 (79.3)	540 (83.7)
≥65	33 (15.3)	16 (14.8)	12 (11.1)	44 (20.7)	105 (16.3)
Gender, n (%)					
Male	54 (25.0)	23 (21.3)	25 (23.1)	42 (19.7)	144 (22.3)
Female	162 (75.0)	85 (78.7)	83 (76.9)	171 (80.3)	501 (77.7)
Race, n (%)					
American Indian or Alaska Native	1 (0.5)	0	0	1 (0.5)	2 (0.3)
Asian	1 (0.5)	0	1 (0.9)	0	2 (0.3)
Black or African American	1 (0.5)	1 (0.9)	2 (1.9)	2 (0.9)	6 (0.9)
White	187 (86.6)	90 (83.3)	90 (83.3)	185 (86.9)	552 (85.6)
Other	26 (12.0)	17 (15.7)	15 (13.9)	25 (11.7)	83 (12.9)
Mean MTX dose, mg/wk (SD)	16.2 (5.2)	15.5 (4.9)	16.2 (4.6)	15.7 (4.6)	15.9 (4.9)
Number of patients with ≥1 prior anti‐TNF treatments, n (%)	14 (6.5)	4 (3.7)	7 (6.5)	16 (7.5)	41 (6.4)
Number of patients with ≥1 concomitant oral steroids for RA, n (%)	127 (58.8)	70 (64.8)	69 (63.9)	137 (64.3)	403 (62.5)
Number of patients with ≥1 NSAIDs for RA, n (%)	128 (59.3)	72 (66.7)	68 (63.0)	126 (59.2)	394 (61.1)

Note

See Figure 1 for explanation of treatment sequences. Percentages based on the number of patients in the Safety Analysis Set with data. Results from the screening visit from the FKB327‐002 study are summarized, apart from weight, which is from week 24 assessment of the FKB327‐002 study.

Abbreviations: F, FKB327; MTX, methotrexate; N, number of patients in the Safety Analysis Set; n, total number of patients with observation; NSAID, nonsteroidal anti‐inflammatory drug; RA, rheumatoid arthritis; RP, reference product (US‐approved); SD, standard deviation; TNF, tumor necrosis factor.

### 3.2 Treatment compliance

Overall, 645 (88.4%) patients continued in Period 2; 10 patients (FKB327, n = 4; RP, n = 6) discontinued treatment in Period 1 but continued participation, whereas 69 patients discontinued Period 1 and declined further participation. The most common reasons for discontinuation were withdrawal of consent (29.4% FKB327% vs 45.7% RP) and occurrence of AEs (41.2% vs 25.7%). In the OLE study, 563 patients received a week 28 dose (final period 2 dose), 517 patients reached and completed the final visit at week 76 (80.2%), and 389 patients (60.3%) received all 39 doses, with a lower proportion of patients receiving all doses in the RP‐F‐F sequence (52.8%). Of the 572 patients who started Period 3, 507 at non‐US sites were switched to use the FKB327 AI starting from week 30; the remaining 65 patients at US sites received FKB327 by prefilled syringe (PFS) due to regulatory requirements.

### 3.3 Immunogenicity comparison between FKB327 and RP

**Figure 2:**
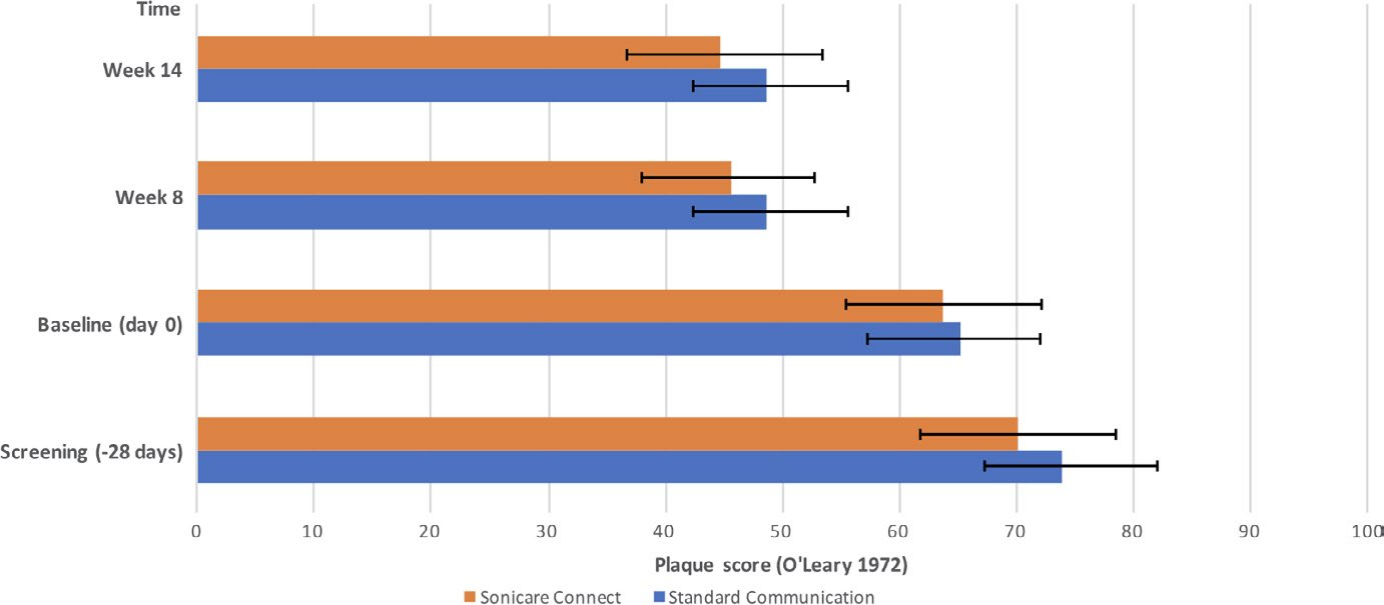
Summary of antidrug antibody development over time. ADA, antidrug antibody; ADA+ve, antidrug antibody positive; F, FKB327; RP, reference product (US‐approved). Week 80 indicates overall; Week 0, beginning of open‐label extension; Week 24, end of Period 2; Week 76, end of Period 3. Percentages are based on the number of patients in the Safety Analysis Set with an assay result obtained each week. See Figure 1 for explanation of treatment sequences. At each sampling point, electrochemiluminescence assays were used to assess the development of ADAs. The proportion of patients with positive ADA status is represented for each treatment sequence at each time point

At the beginning of the OLE study (week 0; Period 2), the proportion of patients with positive ADA status reached the highest during 24 weeks of the preceding DB study, at 61.6% (F‐F), 63.9% (F‐RP), 62.0% (RP‐F), and 58.0% (RP‐RP). At week 76 in the OLE study, the proportion of patients positive for ADAs was 51.1% (F‐F‐F), 54.4% (F‐RP‐F), 48.1% (RP‐F‐F), and 42.5% (RP‐RP‐F; Figure 2). Compared with the beginning of the OLE study, the proportion of patients positive for ADAs was significantly decreased at week 76 in the OLE study for patients in the F‐F‐F (*P* = .0039), RP‐F‐F (*P* = .0018), and RP‐RP‐F (*P* = .0002) groups. During Period 3, the proportion of patients positive for ADAs decreased from week 30 (52.5%) to week 78 (48.4%). The proportion of patients using the AI with positive ADA status did not increase over time and was similar at all time points for both the AI and PFS. At week 76, the proportion of patients positive for ADAs was numerically higher for the AI (48.6%) compared with the PFS (46.7%).

The neutralizing ADA assay was performed on samples that tested positive in the confirmatory ADA assay. At week 30 (end of period 2), 51.9% (F‐F), 50.5% (F‐RP), 45.2% (RP‐F), and 60.0% (RP‐RP) of patients with samples positive for ADAs tested positive for neutralizing ADAs. At all time points during Period 2, similar proportions of patients in each treatment group with samples positive for ADAs tested positive for neutralizing ADAs.

At week 76 (end of Period 3), 51.1% (F‐F‐F), 54.4% (F‐RP‐F), 46.9% (RP‐F‐F), and 42.0% (RP‐RP‐F) of patients tested positive for neutralizing ADAs. During Period 3, 48.0%‐51.8% of patients tested positive for neutralizing ADAs, with 48.0% positive at week 76. During Period 3, 48.4%‐53.3% (48.4% at week 76) of patients using the AI and 42.2%‐46.6% (45.0% at week 76) of patients using the PFS tested positive for neutralizing ADAs.

### 3.4 Safety

Overall, 208 patients (32.2%) experienced a TEAE that was considered to be related to the study drug (Table 2). The most frequently reported treatment‐related TEAEs included nasopharyngitis, bronchitis, and urinary tract infection. The incidence of TEAEs was lower with FKB327 than with the RP (1.707 vs 2.075 events per patient‐year), whereas the incidence of treatment‐emergent serious AEs (TESAEs) was the same for both groups (0.091 events per patient‐year).

**Table 2:** Overall incidence of treatment‐emergent adverse events reported by ≥5 patients receiving either treatment

SOC PT	FKB327 n = 614 673.75 patient‐years		RP n = 321 175.38 patient‐years	
	Patients n (%)	Events n (IR)	Patients n (%)	Events n (IR)
Patients with ≥1 TEAE^1^	411 (66.9)	1150 (1.7)	176 (54.8)	364 (2.1)
Blood and lymphatic system disorders	26 (4.2)	31 (0.05)	10 (3.1)	10 (0.06)
General disorders and administration‐site conditions	29 (4.7)	63 (0.09)	11 (3.4)	22 (0.13)
Injection‐site erythema	6 (1.0)	35 (0.05)	3 (0.9)	9 (0.05)
Infections and infestations	227 (37.0)	375 (0.6)	90 (28.0)	120 (0.7)
Nasopharyngitis	68 (11.1)	77 (0.1)	22 (6.9)	25 (0.1)
Bronchitis	32 (5.2)	35 (0.05)	14 (4.4)	15 (0.09)
Urinary tract infection	30 (4.9)	43 (0.06)	7 (2.2)	8 (0.05)
Upper respiratory tract infection	29 (4.7)	31 (0.05)	9 (2.8)	9 (0.05)
Pharyngitis	24 (3.9)	32 (0.05)	7 (2.2)	7 (0.04)
Latent tuberculosis	17 (2.8)	17 (0.03)	4 (1.2)	4 (0.02)
Sinusitis	11 (1.8)	11 (0.02)	1 (0.3)	1 (0.01)
Pneumonia	7 (1.1)	7 (0.01)	3 (0.9)	3 (0.02)
Investigations	66 (10.7)	103 (0.2)	21 (6.5)	23 (0.1)
*Mycobacterium tuberculosis* complex test positive	13 (2.1)	13 (0.02)	8 (2.5)	8 (0.05)
Alanine aminotransferase increased	11 (1.8)	13 (0.02)	3 (0.9)	3 (0.02)
Aspartate aminotransferase increased	8 (1.3)	11 (0.02)	1 (0.3)	1 (0.01)
Metabolic and nutrition disorders	30 (4.9)	42 (0.06)	14 (4.4)	14 (0.08)
Musculoskeletal and connective tissue disorders	107 (17.4)	155 (0.2)	31 (9.7)	44 (0.3)
Rheumatoid arthritis	37 (6.0)	54 (0.08)	15 (4.7)	18 (0.1)
Nervous system disorders	40 (6.5)	60 (0.9)	11 (3.4)	11 (0.06)
Renal and urinary disorders	20 (3.3)	25 (0.04)	9 (2.8)	13 (0.7)
Respiratory, thoracic, and mediastinal disorders	29 (4.7)	37 (0.06)	8 (2.5)	9 (0.05)
Skin and subcutaneous tissue disorders	24 (3.9)	31 (0.05)	17 (5.3)	20 (0.1)

Note

Percentages are based on the number of patients in the Safety Analysis Set. N for FKB327 includes patients who were randomized to FKB327 in Period 1 and patients who were randomized to the RP and then switched to FKB327 after week 30. Exposure‐adjusted IRs are calculated by dividing the number of events within a given PT or SOC for each treatment by the total number of patient‐years for each treatment. TEAEs are defined as AEs that started or increased in severity after the first study medication administration. Each patient is counted only once within each SOC and PT under the “n (%)” columns but will be counted more than once in the “Events (IR)” columns if more than 1 event within a given SOC or PT occurs. TEAEs were coded using *MedDRA* Version 17.1.

Abbreviations: AE, adverse event; IR, incidence rate (events/patient‐year); MedDRA, Medical Dictionary of Regulatory Activities; N, number of patients in Safety Analysis Set; n, total number of patients with observation; PT, preferred term; RP, reference product; SOC, system organ class; TEAE, treatment‐emergent adverse event.

^a^ IRs for this row are overall IRs based on all TEAEs within each treatment group.

### 3.5 The impact of switching on safety

A similar incidence of TEAEs was reported in the RP‐RP vs the RP‐F sequence (54.9% vs 54.6%) but a lower incidence of TEAEs was reported in the F‐F vs F‐RP sequence (47.7% vs 54.6%), suggesting no consistent effect on the safety of switching treatment. The most common TEAEs and TESAEs in Period 2 were infections and infestations for all treatment sequences. TESAEs were reported by 2.3% (F‐F), 6.5% (F‐RP), 3.3% (RP‐RP), and 4.6% (RP‐F) of patients. Serious infections reported in patients receiving the RP included pneumonia, acute pyelonephritis, bronchitis, appendicitis, and pulmonary mycosis. Patients receiving FKB327 experienced pyelonephritis, pneumonia, sepsis, and appendicitis.

During Period 3, 59.4% of patients experienced at least 1 TEAE, with similar incidences among the F‐RP‐F (61.0%), F‐F‐F (60.3%), and RP‐RP‐F (60.0%) treatment sequences and a lower incidence of TEAEs in the RP‐F‐F treatment sequence (54.8%; Table 3). The most frequently reported TEAEs during Period 3 were nasopharyngitis, urinary tract infection, bronchitis, and upper respiratory infection. TESAEs were reported by 33 patients (5.8%), and ranged from 4.2% (F‐F‐F) to 8.0% (F‐RP‐F).

**Table 3:** Treatment‐emergent adverse events by treatment sequence in Period 3

	F‐F‐F N = 189 n (%)	F‐RP‐F N = 100 n (%)	RP‐F‐F N = 93 n (%)	RP‐RP‐F N = 190 n (%)	Total N = 572 n (%)
Deaths	0	0	1 (1.1)	1 (0.5)	2 (0.3)
Treatment‐emergent deaths	0	0	1 (1.1)	1 (0.5)	2 (0.3)
Patients with ≥1 TEAEs	114 (60.3)	61 (61.0)	51 (54.8)	114 (60.0)	340 (59.4)
Patients with ≥1 severe TEAEs	2 (1.1)	8 (8.0)	4 (4.3)	5 (2.6)	19 (3.3)
Patients with ≥1 treatment‐related TEAEs	43 (22.8)	24 (24.0)	22 (23.7)	37 (19.5)	126 (22.0)
Patients who prematurely discontinued treatment due to a TEAE	4 (2.1)	5 (5.0)	6 (6.5)	10 (5.3)	25 (4.4)
Patients who prematurely discontinued treatment due to a TESAE	2 (1.1)	2 (2.0)	3 (3.2)	3 (1.6)	10 (1.7)
Patients who had a treatment interruption due to a TEAE	14 (7.4)	5 (5.0)	8 (8.6)	9 (4.7)	36 (6.3)
Patients who had a treatment interruption due to a TESAE	2 (1.1)	0	2 (2.2)	2 (1.1)	6 (1.0)
TESAEs, n	8	11	11	15	45
Patients with ≥1 TESAEs	8 (4.2)	8 (8.0)	6 (6.5)	11 (5.8)	33 (5.8)
Patients with ≥1 SAEs	8 (4.2)	8 (8.0)	6 (6.5)	11 (5.8)	33 (5.8)

Note

Percentages are based on the number of patients in the Safety Analysis Set who entered Period 3. Death was defined as the fatal outcome of an (S)AE. SAEs were defined as AEs that were fatal, life‐threatening, or required or prolonged inpatient treatment; resulted in persistent or significant disability or incapacity; were a congenital anomaly or birth defect; or were medically important events that may have jeopardized the patient. TEAEs were defined as AEs that started or increased in severity after the first study medication administration. Severe TEAEs were defined as SAEs occurring or increasing in severity after the first dose of study medication was taken. Related TEAEs were defined as TEAEs for which the relationship to study medication was recorded as “Related,” “Possibly related,” or missing. AEs were counted under the treatment arm and period in which the event started.

Abbreviations: AE, adverse event; F, FKB327; N, number of patients in Safety Analysis Set; n, total number of patients with observation; RP, reference product (US‐approved); SAE, serious adverse event; TEAE, treatment‐emergent adverse event; TESAE, treatment‐emergent serious adverse event.

A similar proportion of patients from most treatment sequences experienced TEAEs leading to discontinuation during Period 3 (F‐RP‐F, 5.0%; RP‐F‐F, 6.5%; RP‐RP‐F, 5.3%); however, only 2.1% of patients in the F‐F‐F sequence experienced TEAEs leading to discontinuation (Table 3). By contrast, fewer patients in the F‐RP‐F (5.0%) and RP‐RP‐F (4.7%) sequences reported TEAEs that resulted in treatment interruption.

The incidence of hypersensitivity or anaphylaxis (TEHAE) was low among all patients, with a numerically lower incidence in FKB327‐treated patients than RP‐treated patients (0.009 vs 0.046, respectively). The incidence of TEHAE was slightly numerically higher in ADA‐positive patients in both groups (5 events [0.015] vs 6 events [0.069]) compared with ADA‐negative patients (1 event [0.003] vs 2 events [0.022]). Furthermore, the incidence of injection‐site reactions was low in both groups (0.080 vs 0.059, respectively), and was not higher in either group for ADA‐positive patients (5 events [0.015] vs 5 events [0.058]) compared with ADA‐negative patients (35 events [0.103] vs 9 events [0.101]). During Period 2, injection‐site reactions were reported for ADA‐negative patients treated with F‐F (n = 3; 2.9%), RP‐RP (n = 3; 2.9%), and RP‐F (n = 1; 1.8%), and for ADA‐positive patients treated with F‐RP (n = 2; 3%). During Period 3, injection‐site reactions were reported for ADA‐negative patients treated with F‐F‐F (n = 2; 2.4%), F‐RP‐F (n = 1; 2.4%), and RP‐F‐F (n = 1; 2.0%), and for ADA‐positive patients treated with F‐F‐F (n = 1; 1.0%) and F‐RP‐F (n = 1; 1.9%). No relationship between these AEs and ADA titer category was apparent.

### 3.6 The impact of switching on pharmacokinetics

**Figure 3:**
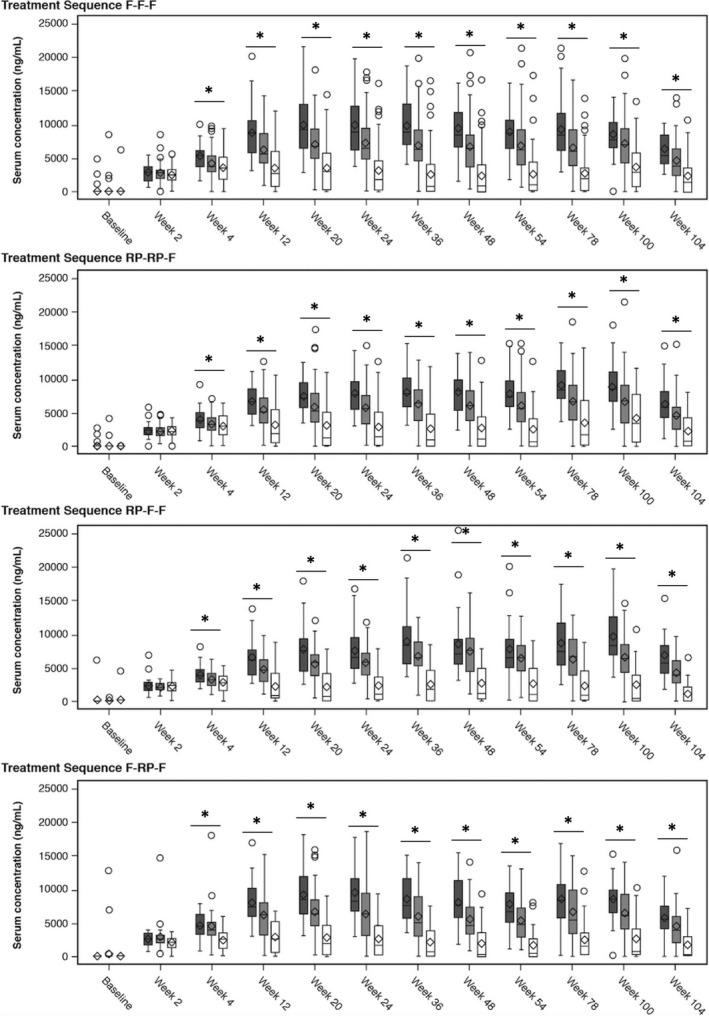
Drug serum trough concentrations and ADA status over time. ADA, antidrug antibody; DB, double‐blind; F, FKB327; OLE, open‐label extension; RP, reference product (US‐approved). Baseline indicates the beginning of the DB study; Week 24, beginning of OLE; Week 54, end of Period 2, OLE; Week 76, end of Period 3, OLE; Week 100, Week 76, OLE. See Figure 1 for full explanation of treatment sequences. Low, less than or equal to the lower quartile (0.0625); moderate, between the lower and upper quartile; high, greater than or equal to the upper quartile (17 600). Circles indicate outlier points; rectangles, the upper and lower quartiles; horizontal line inside rectangle, median; vertical lines extending from rectangles, highest and lowest values; diamonds, means. *Indicates time points where an analysis of variance model including maximum ADA titer category as the only covariate had a significant (*P* < .05) overall *F* test. Validity of assumptions was evaluated via diagnostic plots (histograms, scatterplots, and quantile‐quantile plots)

Mean serum trough drug concentrations at week 0 (week 24 overall; start of Period 2) were higher among patients receiving FKB327 in Period 1 (F‐F, 6500 ng/mL; F‐RP, 6000 ng/mL; RP‐F, 5170 ng/mL; RP‐RP, 5720 ng/mL). In all treatment sequences, the interindividual variability was high; however, the mean serum trough drug concentration was generally stable between week 0 (week 24 overall; start of Period 2) and week 30 (week 54 overall; start of Period 3) for the F‐F‐F, RP‐F‐F, and RP‐RP‐F sequences (6000 ng/mL, 5730 ng/mL, and 5750 ng/mL, respectively; Figure 3). A slight downward shift was reported for the F‐RP‐F sequence. Mean serum trough drug concentrations increased slightly in all sequences to week 76 (week 100 overall); the interindividual variability was high in all sequences throughout Period 3. At week 76, the mean serum trough drug concentrations were 6460 ng/mL (F‐F‐F), 5900 ng/mL (F‐RP‐F), 6070 ng/mL (RP‐F‐F), and 6730 ng/mL (RP‐RP‐F). Participants receiving the F‐RP‐F sequence exhibited a slightly lower mean serum trough drug concentration and a slightly higher proportion of patients positive for ADAs.

Examining serum concentrations by maximum ADA titer over time, significant differences were observed among patients with low (≤lower quartile; 0.0625), moderate (between the lower and upper quartile), and high (≥upper quartile; 17 600) ADA titer from week 4 through week 104 of the entire study (Figure 3).

To evaluate PK in patients who switched treatments, ratios of geometric least squares means at all time points in Period 2 were estimated. Patients who received the RP in Period 1 but switched to FKB327 were estimated to have up to 11% higher drug exposure than those who continued receiving the RP during Period 2. Patients who received FKB327 in Period 1 but switched to the RP were estimated to have a 20%‐29% lower drug exposure than those who continued receiving the RP during Period 2 (Figure 3).

## 4 DISCUSSION

This was the final analysis of the 2‐year, combined DB and OLE studies designed to compare the safety, efficacy, and immunogenicity of FKB327 with the RP in patients with moderate‐to‐severe RA, for which the preliminary analysis of data through 54 weeks has been previously published.^9^ The current analysis focused on immunogenicity over long‐term use (out to 2 years) to support the biosimilarity of FKB327 to the RP and to assess the impact of switching on safety and PK. Assessing immunogenicity is important, because biologic therapies have unique structures that can induce immune responses, which may lead to the development of therapy‐limiting AEs.^5^ Furthermore, ADAs have been implicated as contributors to increased risk for AEs and treatment failure in patients who are treated with biologics.^6^

ADA formation can lead to diminished treatment efficacy via different mechanisms.^6^ Neutralizing ADAs can block the binding of the therapeutic agent to its target, which reduces treatment efficacy. In addition, both neutralizing and non‐neutralizing ADAs can bind to the therapeutic agent, which can result in the formation of immune complexes that are subsequently cleared from the circulation, resulting in reduced drug half‐life. In a study evaluating adalimumab‐specific ADAs, at least 98% had neutralizing capabilities.^10^ Dimer‐sized immune complexes between ADAs and adalimumab have been detected in patients 2 weeks postinjection, suggesting that these immune complexes are not cleared rapidly from the circulation.

Not all patients will respond favorably to biological treatment; some do not exhibit any response, whereas others initially exhibit a response but demonstrate a loss of response over time despite administration of higher doses of the drug and/or more frequent drug administration.^11^ ADA formation contributes to this observed loss of response through decreased serum levels of biologicals via increased clearance and inactivation, which ultimately contributes to premature therapy termination.^12^ Therefore, some patients exhibit subtherapeutic levels of drugs, which decreases efficacy. By identifying these patients via monitoring of ADA formation and drug serum trough levels, appropriate treatment guidance can be provided.

Tolerance induction during repeated administration of biologics may be considered. Other biosimilar studies reported maintained immunogenicity during long‐term treatment, with low ADA development. In our study, ADA positivity reached a near plateau at the beginning of Period 2. There was a low level of newly developed ADA in the OLE study, with ADA levels maintained in all sequences during long‐term treatment. A slight decrease of ADA positivity without an increase of ADA titer at each visit was considered potential immune tolerance during long‐term treatment with a TNF inhibitor, but it is unknown because drug exposure of subcutaneous adalimumab is not high.

Patients with RA receiving anti‐TNF agents in combination with MTX, azathioprine, hydrocortisone, or mercaptopurine have exhibited a reduced frequency of ADA formation,^13^, ^14^, ^15^ which may contribute to the lower ADA levels seen over time. Alternatively, factors related to the measurement of ADA may influence these results. Many assays used to detect ADA predominantly detect free ADA, highlighting complications with drug interference due to the presence of high levels of therapeutic antibody in serum samples from patients.^16^ Assays sensitive to the effects of drug interference are designed to detect ADA only if they are present in higher amounts than the circulating drug. Because many patients receive continuous treatment with therapeutic monoclonal antibodies, drug interference can contribute to false‐negative readings or to the underestimation of the total ADA amount present in the sample.^17^ Drug interference influence is variable between assays, which also contributes to difficulties detecting ADA. Assays have been developed to measure both bound and free ADA in the presence of the active drug. The pH‐shift anti‐idiotype antigen‐binding test is one assay that enables detection of ADAs independent of drug levels.^18^

The ADA assay used in the current study includes an acid dissociation step to improve drug tolerance for multiple‐dosing studies. In the validation study, we confirmed an assay sensitivity of 9.9 ng/mL and ADA detected in the presence of 50 μg adalimumab/mL of serum, which covered the serum concentration of adalimumab in clinically repeated doses. Thus, there is no impact of the ADA detection method on the proportion of ADA‐positive patients.

There is a potential decrease of ADA‐positive patients at each visit if many of these patients dropped out of the study. We have investigated the ADA‐positive ratio among those who dropped out of the study because it is possible that ADA positivity may cause reduced treatment efficacy. The ADA‐positive ratio among patients who dropped out of the study was 56.59%, which was not a major difference in the ADA‐positive ratio in the overall patient population. Newly developed ADA in patients who dropped out of the study only occurred in 1 patient in the RP‐RP‐F group. Thus, it was not expected that many ADA‐positive patients dropped out of the study and caused a decrease of ADA‐positive patients at each visit.

FKB327 and the RP showed equivalent immunogenicity over long‐term administration. Importantly, single and double switching between treatments did not have any clinically relevant effects on ADA development. In the current study, the proportion of patients positive for ADAs at week 76 was similar across all treatment sequences. These findings of similar immunogenicity support our previous study in healthy individuals, which reported detectable ADAs in most treated subjects, with similar results between FKB327 and the RP.^4^

Switching between FKB327 and the RP, either via a single switch (F‐F‐RP or RP‐RP‐F) or a double switch (F‐RP‐F), had no meaningful impact on immunogenicity. At the end of Period 2, most patients with samples positive for ADAs tested positive for neutralizing ADAs. During Period 3, no increases were reported for positive ADA status, ADA titer, or positive neutralizing ADA status through week 76 in any treatment sequence, and ADA levels were equivalent for patients treated with FKB327 and the RP.

Regarding the decrease in the proportion of patients testing positive for ADAs throughout the study, it has been shown that patients treated with natalizumab or adalimumab exhibited decreasing ADA levels over time, which was suggestive of immune tolerance induction.^7^ One study reported approximately one‐third of adalimumab‐treated patients became tolerant over the treatment period.^6^

The current results support findings from a review of 53 switching studies, which demonstrated no unexpected safety findings post‐switch.^19^ The current results also extend the findings of the preliminary analysis, which included data through the first 54 weeks,^9^ with no demonstrated effect of switching between FKB327 and RP in terms of safety or immunogenicity. This is supported by findings from the current, final analysis, which extended to 2 years of treatment and follow‐up. The current study adds to the existing body of literature, demonstrating that patients who received FKB327 long‐term throughout the study exhibited similar safety and immunogenicity results compared with patients who switched between the biosimilar and the RP.

Importantly, in this study, the incidence of TESAEs was low and a similar incidence was reported for patients treated with FKB327 and the RP. These findings further support our previous phase 1 investigation in healthy individuals, which reported that 53.9% of patients experienced treatment‐related TEAEs, including a single subject reporting a severe AE with FKB327 and the RP.^4^ Importantly, AEs were similar between FKB327 and the RP. Furthermore, no appreciable differences were observed among the treatment sequences, with similar AEs reported for patients experiencing no switch, a single switch, and a double switch. Taken together, these findings highlight that there were no unexpected AEs reported in patients treated with FKB327, and that switching between the RP and FKB327 revealed no new or more frequent safety signals.

In addition, no meaningful differences in the safety profile of those who used the AI in Period 3 were reported. Switching between delivery of FKB327 via a PFS to an AI had no effect on serum trough drug concentrations or ADA status. A recent study demonstrated that patients randomized to receive an adalimumab biosimilar via an AI or PFS exhibited similar immunogenicity, independent of injection site.^20^

Other studies have supported the use of anti‐TNF biosimilars. A phase 3 trial investigating a rituximab biosimilar in patients with RA demonstrated that long‐term use of the biosimilar to 72 weeks was well tolerated and effective.^21^ In this study, patients who underwent a single switch from the RP to the biosimilar exhibited similar results in terms of efficacy, pharmacodynamics, immunogenicity, and safety. A phase 3 study of an infliximab biosimilar in patients with moderate‐to‐severe RA demonstrated similar safety, efficacy, and immunogenicity of the biosimilar compared with the RP to 54 weeks.^22^ Furthermore, a single treatment switch at week 30 did not affect these outcomes. In a DB equivalence study, an adalimumab biosimilar exhibited similar safety, efficacy, and immunogenicity to the RP, with no impact of a single treatment switch at week 24 and follow‐up to 58 weeks.^23^ These findings support our current data, improving our knowledge on long‐term safety and immunogenicity of the adalimumab biosimilar FKB327, demonstrating similar safety and immunogenicity compared with the RP, with a 6‐month switching interval and 2 years of follow‐up.

By filling the information gap related to long‐term immunogenicity, safety, and PK, these data will help inform clinician decision making regarding switching from the RP to FKB327, and may result in increased patient access to adalimumab treatment.

## CONFLICT OF INTEREST

Dr Alten has received consultant fees from Mylan Inc, and has been a paid speaker for Mylan Inc Dr Markland reports personal fees from Fujifilm Kyowa Kirin Biologics Co. Ltd. during the conduct of the study, as well as personal fees from Fujifilm Kyowa Kirin Biologics Co. Ltd. outside the submitted work. Dr Boyce has nothing to disclose. Mr Kawakami is an employee of Fujifilm Kyowa Kirin Biologics Co. Ltd. Dr Muniz is an employee and shareholder of Mylan Inc Dr Genovese has received consultant fees from Fujifilm Kyowa Kirin Biologics Co. Ltd., including for the design of this trial.

## AUTHOR CONTRIBUTIONS

Dr Alten initiated the study design and helped with implementation; provided statistical expertise in clinical trial design and conducted the primary statistical analysis; contributed to refinement and development of the manuscript; and approved the final manuscript. Dr Markland contributed to the study design and helped with implementation; provided medical review of the clinical study data; contributed to the refinement and development of the manuscript; and approved the final manuscript. Dr Boyce contributed to the refinement and development of the manuscript and approved the final manuscript. Mr Kawakami initiated the study design and helped with implementation; provided clinical pharmacology expertise in clinical trial design and reviewed the study results from clinical pharmacology perspectives; contributed to refinement and development of the manuscript; and approved the final manuscript. Dr Muniz contributed to the refinement and development of the manuscript and approved the final manuscript. Dr Genovese initiated the study design and helped with implementation; contributed to the analysis and interpretation of the data; contributed to development and refinement of the manuscript; and approved the final manuscript. All authors are fully responsible for all content and editorial decisions and received no financial support or other form of compensation related to the development of this manuscript. All authors had final approval of the manuscript and are accountable for all aspects of the work in ensuring the accuracy and integrity of this manuscript.

## CLINICAL TRIALS REGISTRATION

FKB327‐002 DB study –NIH US National Library of Medicine, NCT02260791, https://clinicaltrials.gov/ct2/show/NCT02260791/EudraCT Number: 2014‐000109‐11. FKB327‐003 OLE study – NIH US National Library of Medicine, NCT02405780, https://clinicaltrials.gov/ct2/show/NCT02405780/EudraCT Number: 2014‐000110‐61.

## Data Availability

The data that support the findings of this study are available from the corresponding author upon reasonable request.
